# Supplementary material for: Germline mutation analyses of malignant ground glass opacity nodules in non-smoking lung adenocarcinoma patients
Source: PeerJ. 2021 Aug 31;9:e12048. doi: 10.7717/peerj.12048 (PMC8415279; doi:10.7717/peerj.12048)
Supplement: Supplemental Information 1 — Detailed description of methods used to screen candidate variants [file peerj-09-12048-s001.docx]

### Methods used to screen candidate variants are described below.

### Trimmomatic-0.36 ([Bolger, Lohse, & Usadel, 2014](#_ENREF_4)) was used as quality control for raw data and to remove adapters. Clean sequence reads were aligned to the human reference genome (GRCh37/b37 assembly) using Burrows-Wheeler Aligner software (version 0.7.10) ([Li & Durbin, 2009](#_ENREF_16)). Picard (version 2.9.2, Broad Institute, Boston, MA, USA) was used to remove duplicates. Variant detection was performed using HaplotypeCaller in the Genome Analysis Toolkit 3.4 (www.broad institute.org/gatk) ([DePristo et al., 2011](#_ENREF_7)). Variants were annotated using InterVar database.

Manual screening process is as follows:

1. Variants tagged as B/LB and not P/LP by InterVar were excluded；
2. Variants tagged as P/LP by InterVar was included;
3. High frequency variants (0.5%) in 1000G databse;
4. Variants not on the hereditary gene panel (Off-candidate) were excluded;
5. Variants on the gene were included;

After the above process, we continue to analyze the candidate variants and the process is as follows:

1. Variants with MAF (Minor allele frequency) that is no less than 0.5% was excluded;
2. Unreliable sequencing results, like reads less than 10 or poly A, were excluded;
3. Non-cancer-related genes (searched in google scholar or Pubmed) were excluded;
4. Variants tagged by ACMG as B/LB were excluded;
5. Synonymous mutations or not in CDS were excluded;

The gene panel was designed referring to the genes from the following source

https://www.genome.jp/dbget-bin/www_bget?hsa04010

http://www.ongene.bioinfo-minzhao.org/download.html

https://bioinfo.uth.edu/TSGene/download.cgi

https://www.cancerhotspots.org/#/home

After all the previous steps, we obtained disease-causing candidate variants.
