# Supplementary material for: Germline mutation analyses of malignant ground glass opacity nodules in non-smoking lung adenocarcinoma patients
Source: PeerJ. 2021 Aug 31;9:e12048. doi: 10.7717/peerj.12048 (PMC8415279; doi:10.7717/peerj.12048)

All patients were closely followed from the past 6 months to 3 years. The size of the GGO nodules either increased or the nodule density increased or the solid components of nodules increased. All patients expressed their strong will to have a surgery. Surgery is the result of comprehensive considerations from the clinical point. All the process strictly follows the guidelines for the clinical diagnosis and treatment of lung cancer.

Patient ID	Pathology	Tumour size (cm)	Tumour location
WL-1	LUAD†, invasive	1.2	right upper lobe
WL-2	LUAD†, AIS*	0.6	left upper lobe
WL-3	Atypical adenomatous hyperplasia	0.5	right upper lobe
WL-4	LUAD†, invasive	1.5	left upper lobe
WL-5	LUAD†, minimally invasive	1.0	right upper lobe
WL-6	LUAD†, minimally invasive	0.6	left upper lobe
WL-7	LUAD†, invasive	2.0	right upper lobe
WL-8	LUAD†, invasive	2.0	right lower lobe
WL-9	LUAD†, AIS*	0.8	right upper lobe
WL-10	LUAD†, AIS*	0.7	right upper lobe
WL-11	LUAD†, AIS*	0.6	right lower lobe
WL-12	LUAD†, AIS*	0.8	right upper lobe
WL-13	LUAD†, minimally invasive	0.7	right lower lobe

† lung adenocarcinoma; * adenocarcinoma in situ; **ground glass opacity;

WL-1 CT (left) and IHC (right)

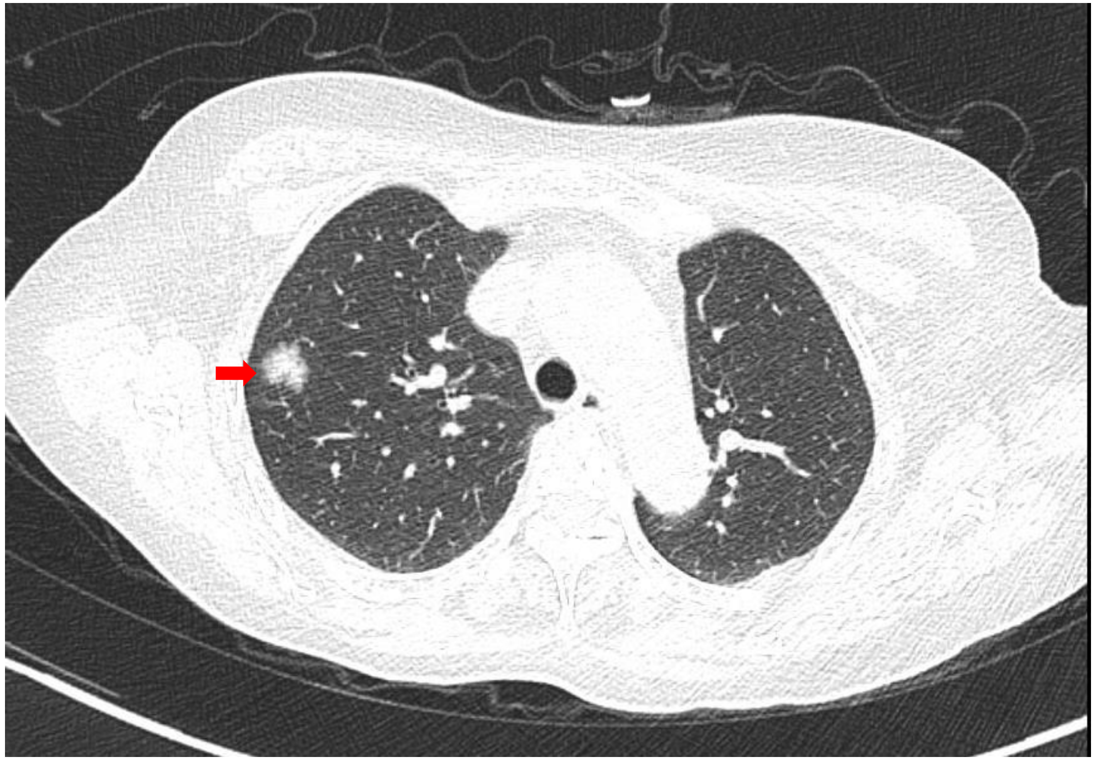

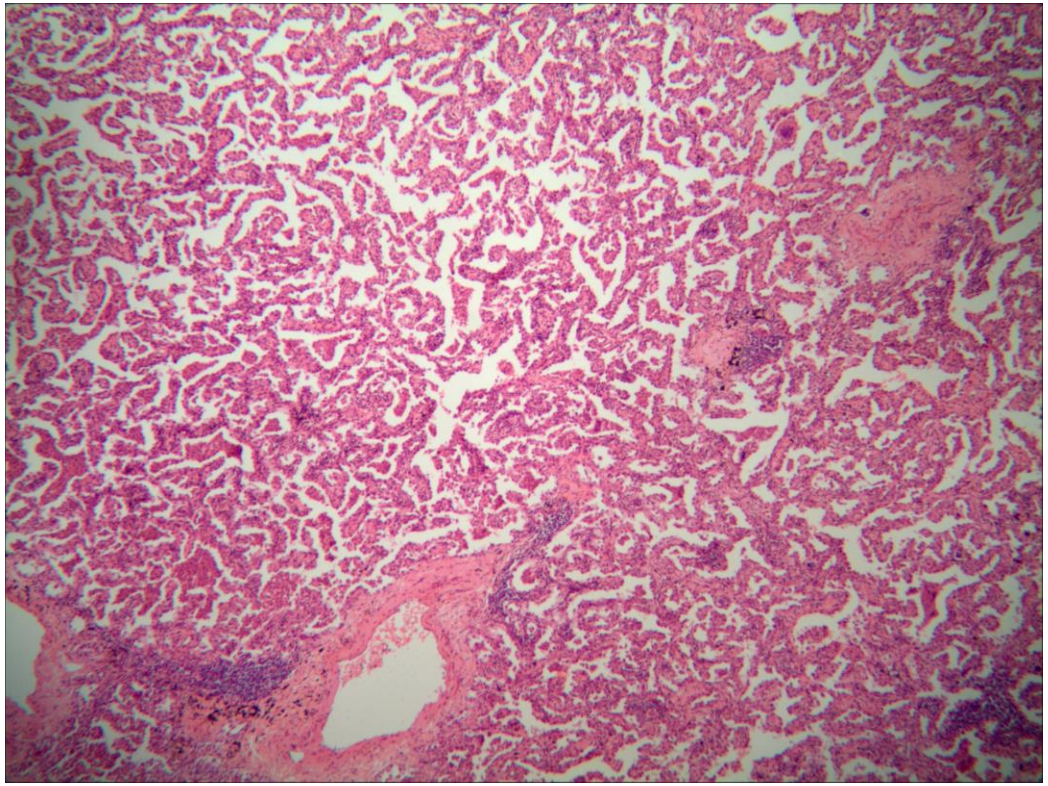

WL-2 CT (left) and IHC (right)

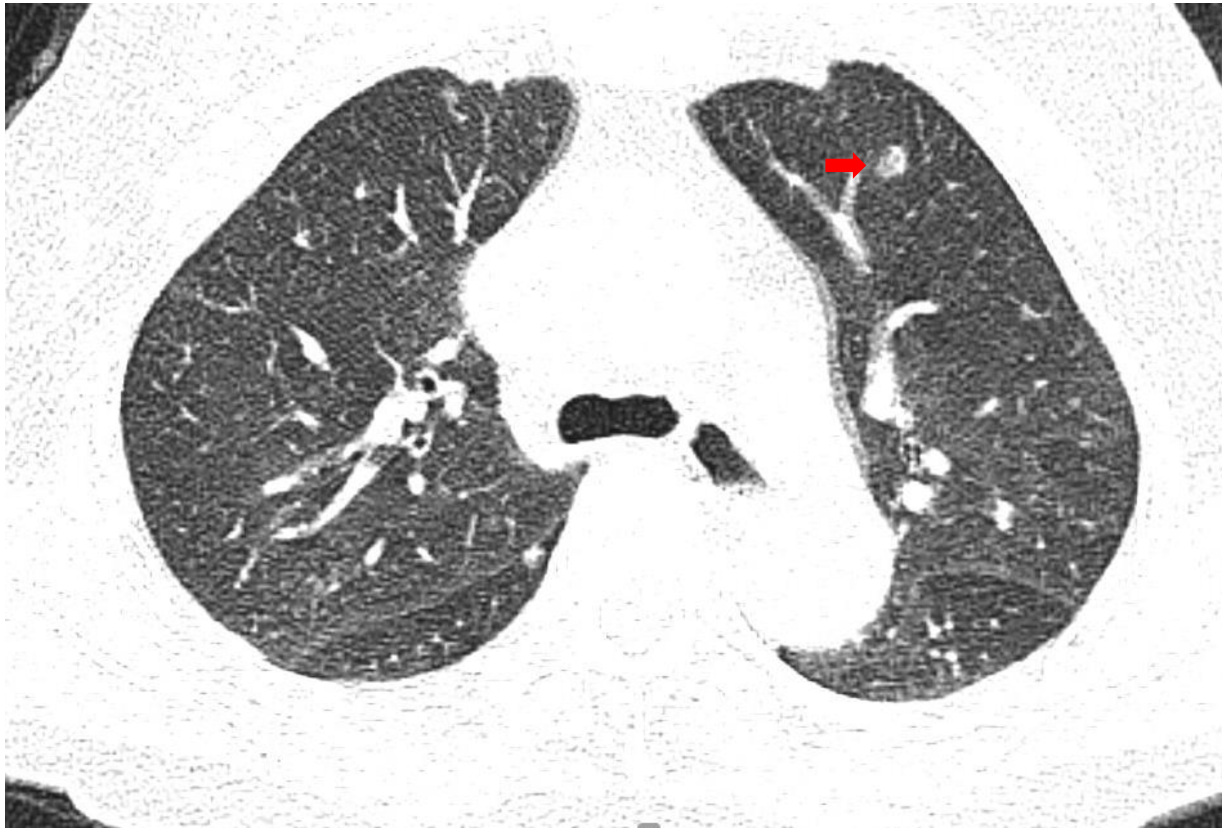

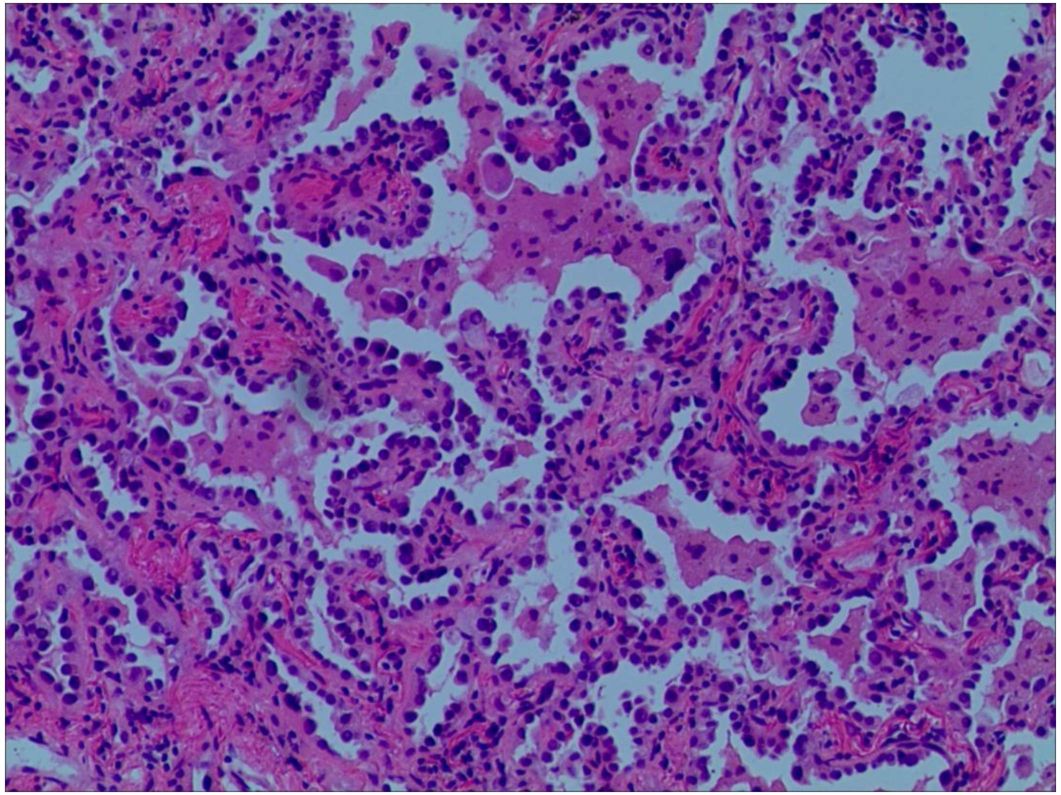

WL-3 CT (left) and IHC (right)

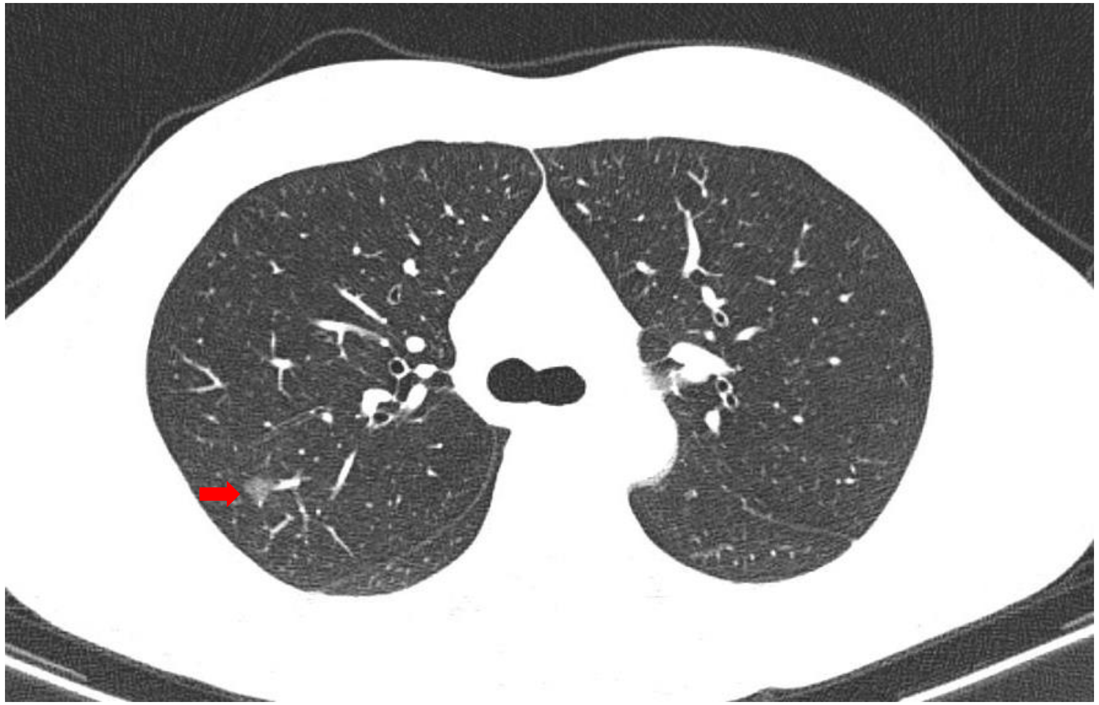

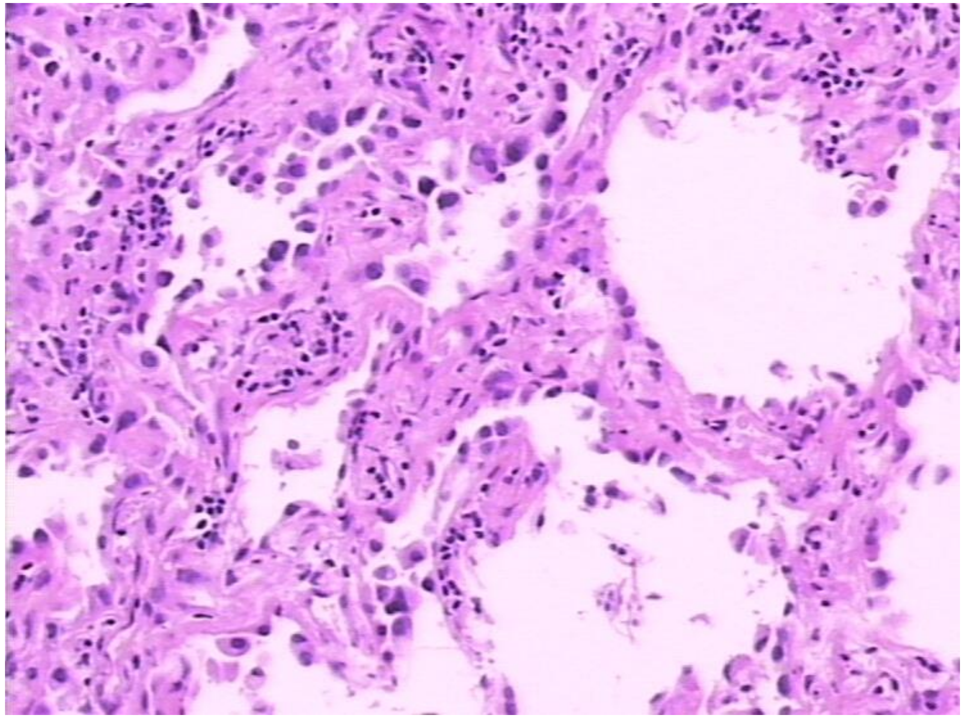

WL-4 CT (left) and IHC (right)

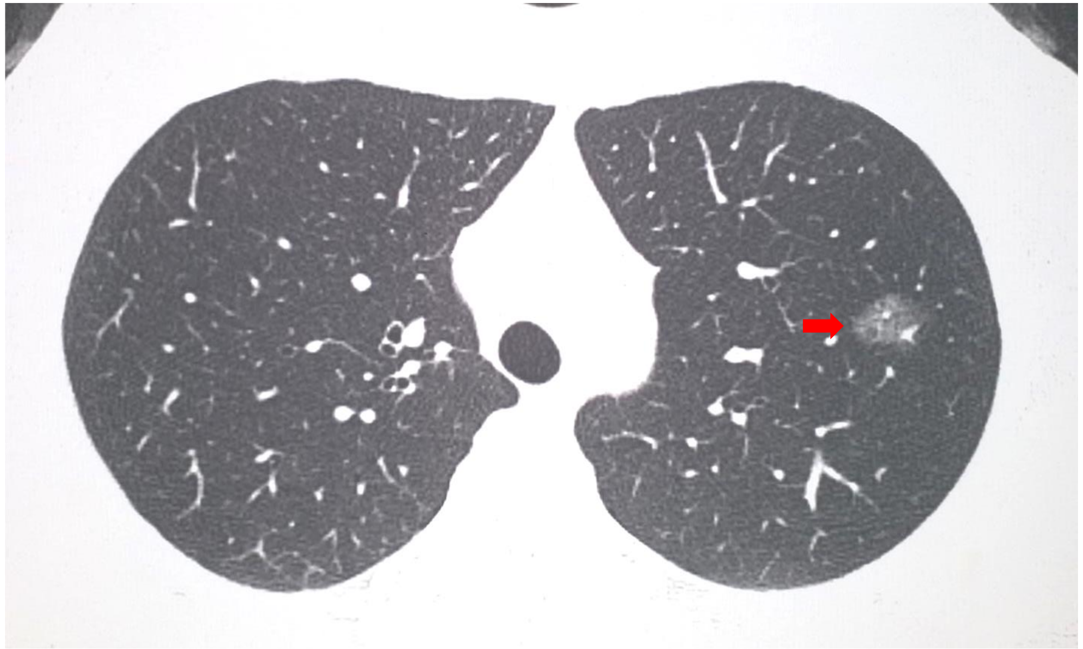

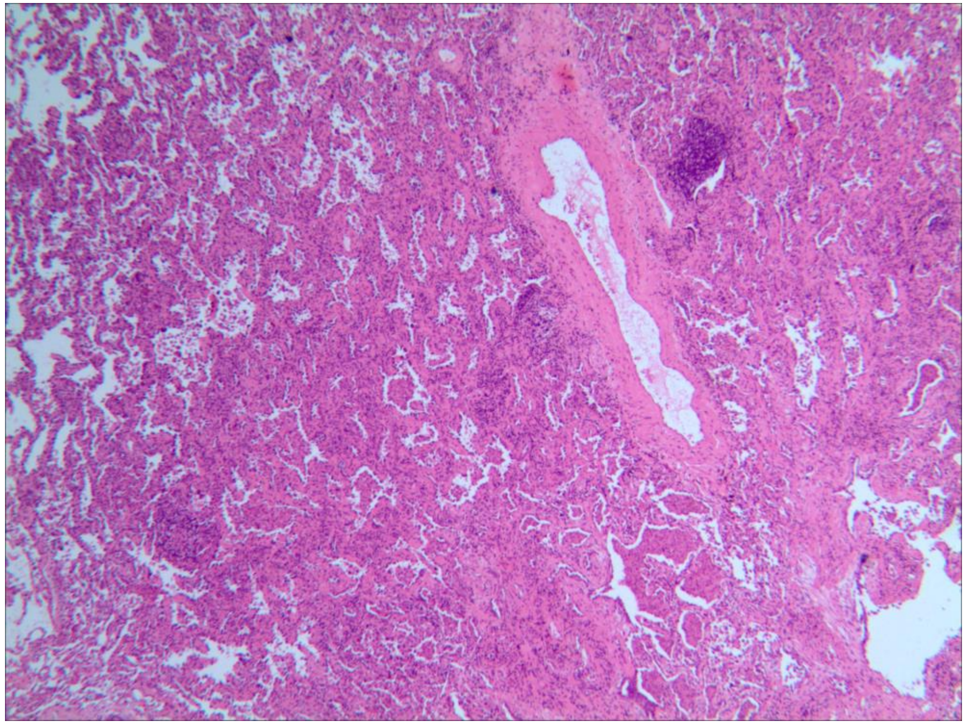

WL-5 CT (left) and IHC (right)

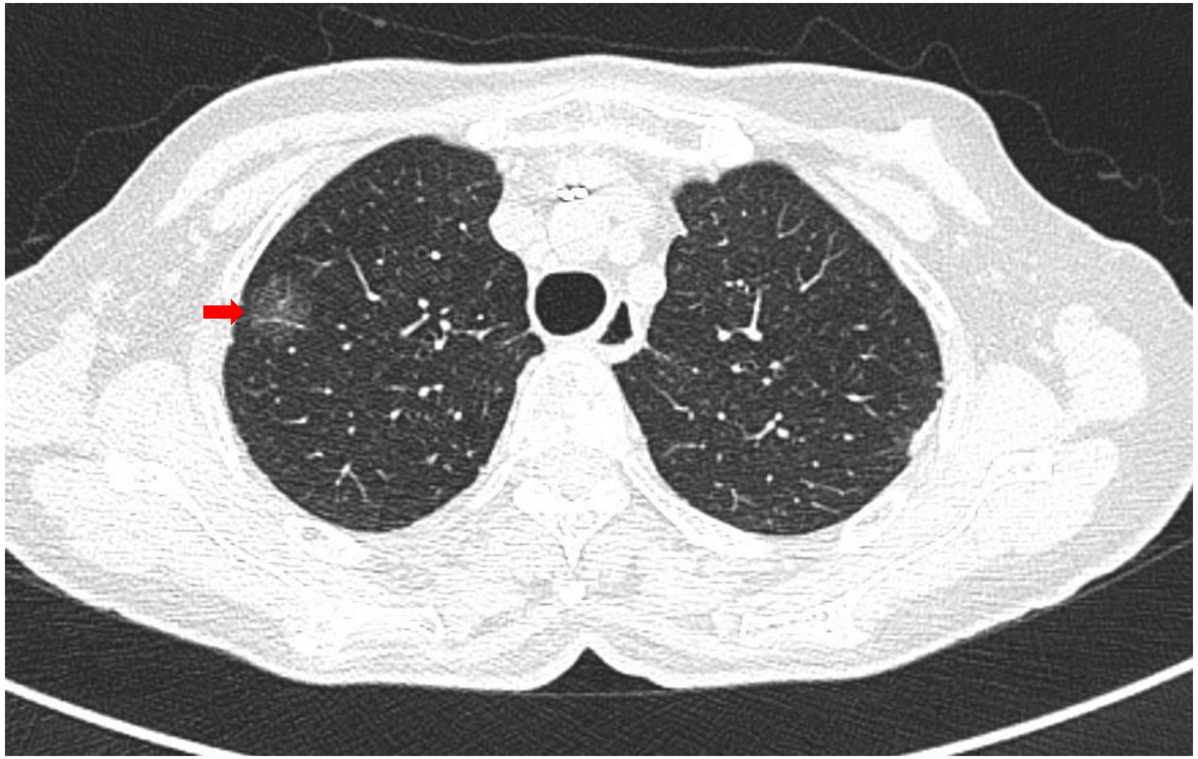

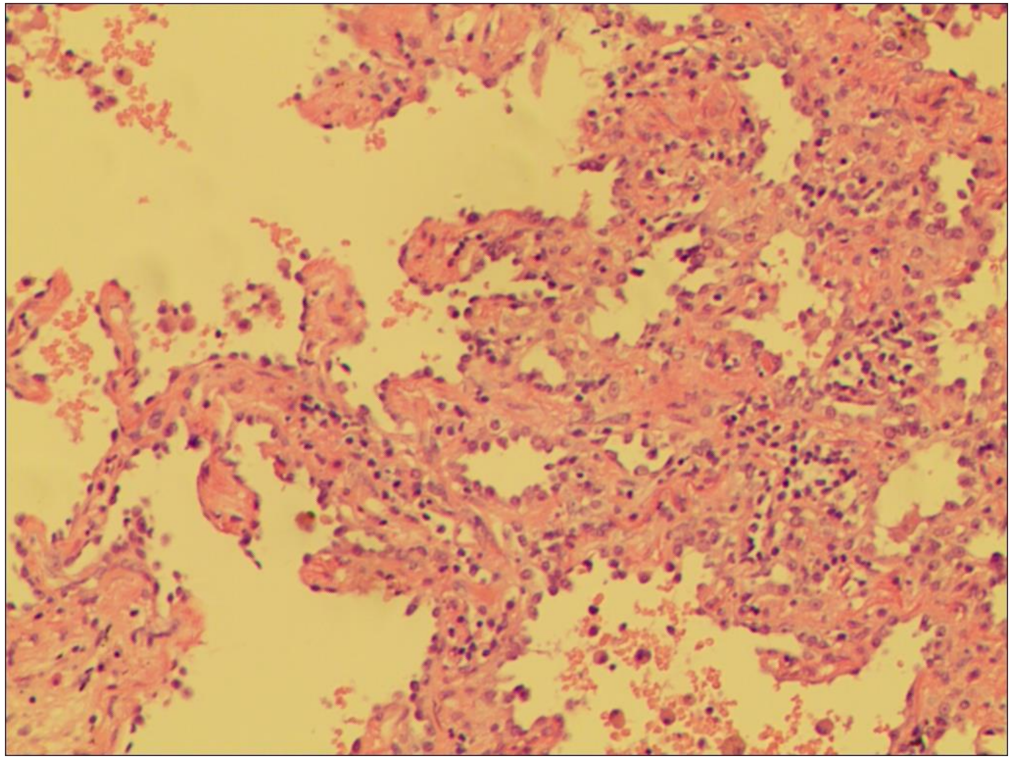

WL-6 CT (left) and IHC (right)

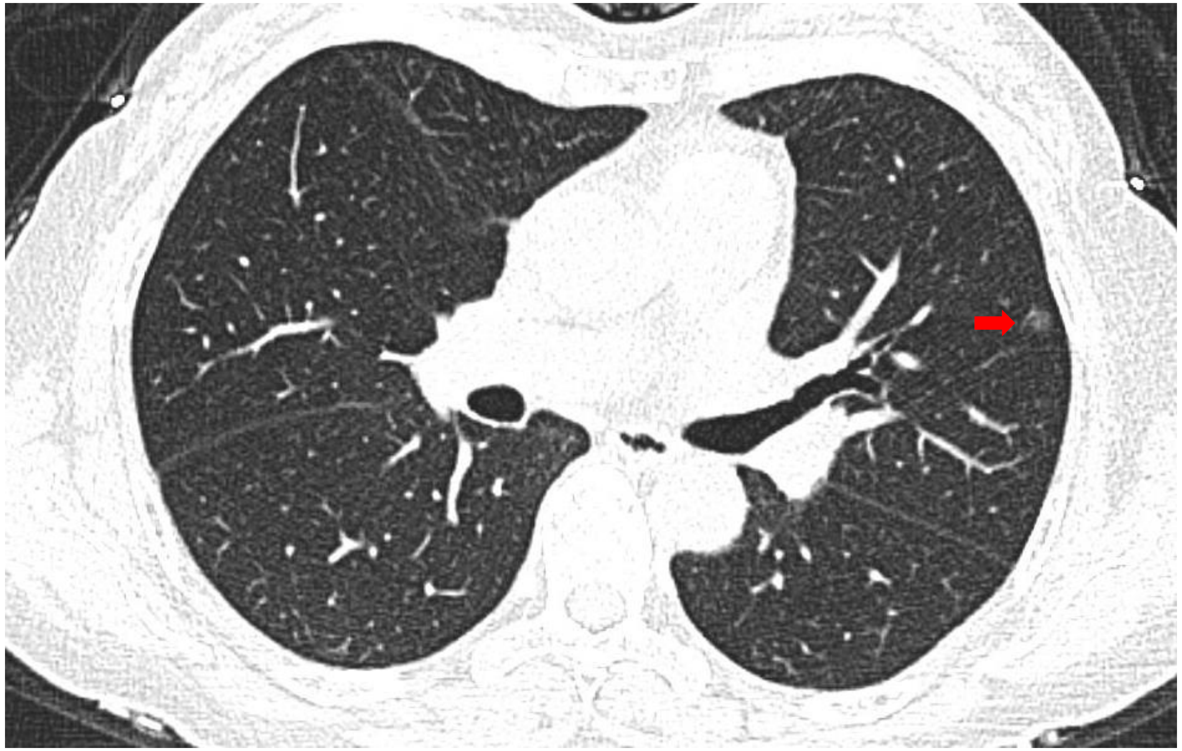

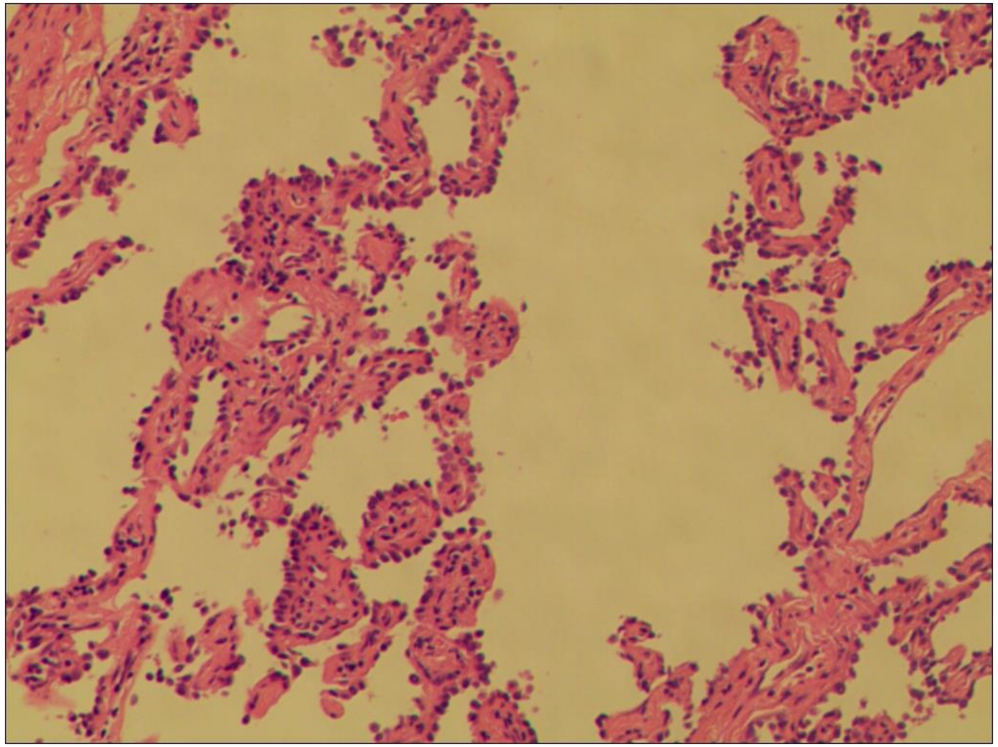

WL-7 CT (left) and IHC (right)

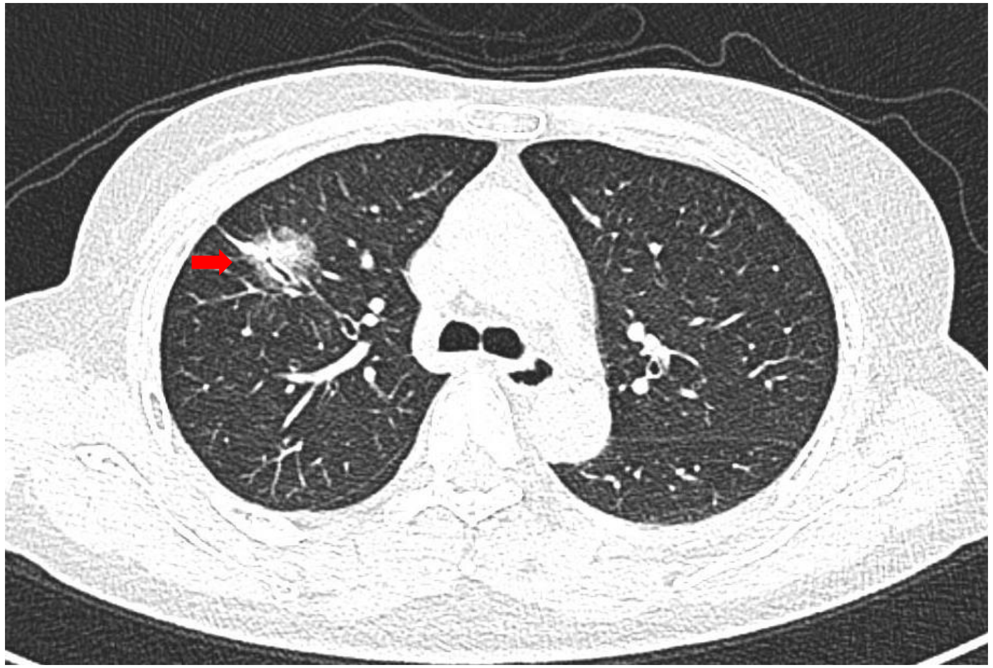

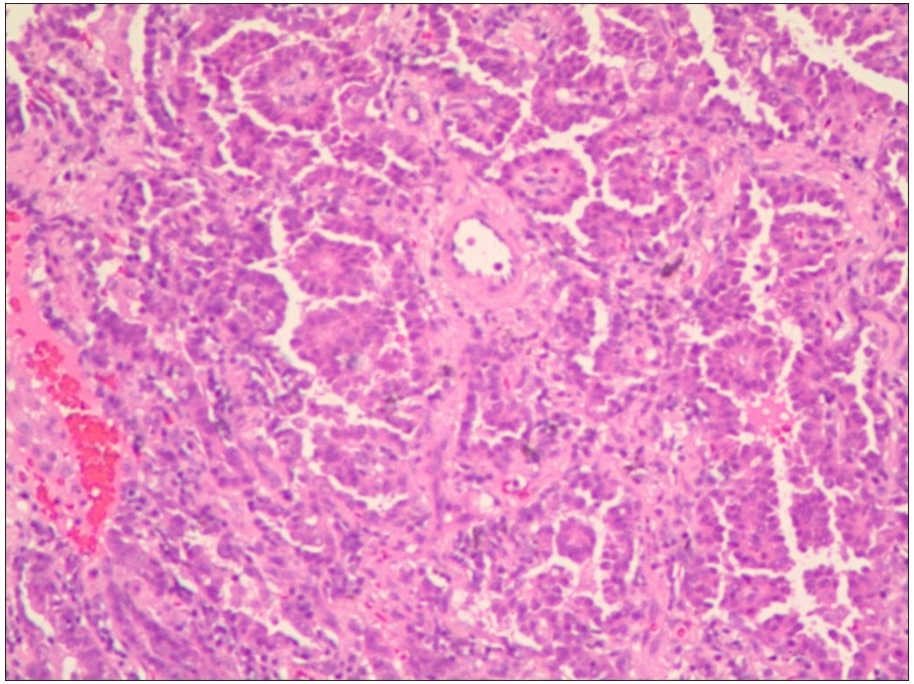

WL-8 CT (left) and IHC (right)

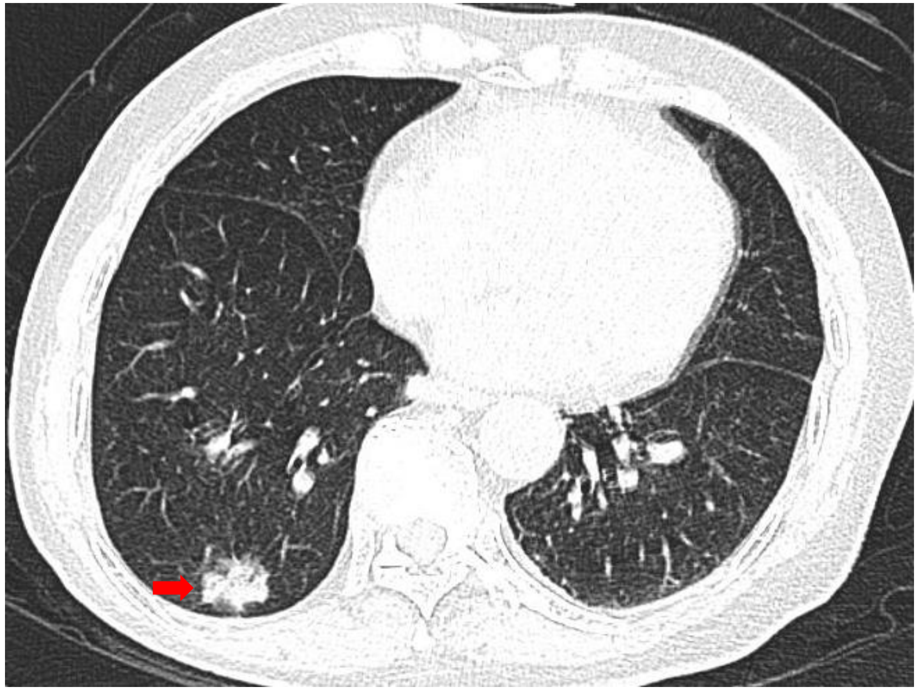

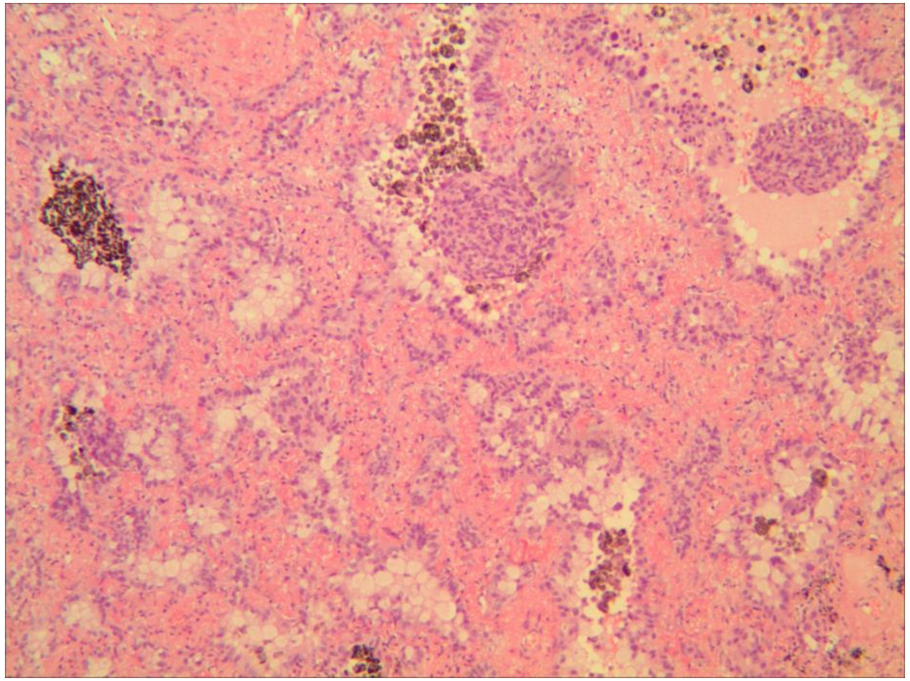

WL-9 CT (left) and IHC (right)

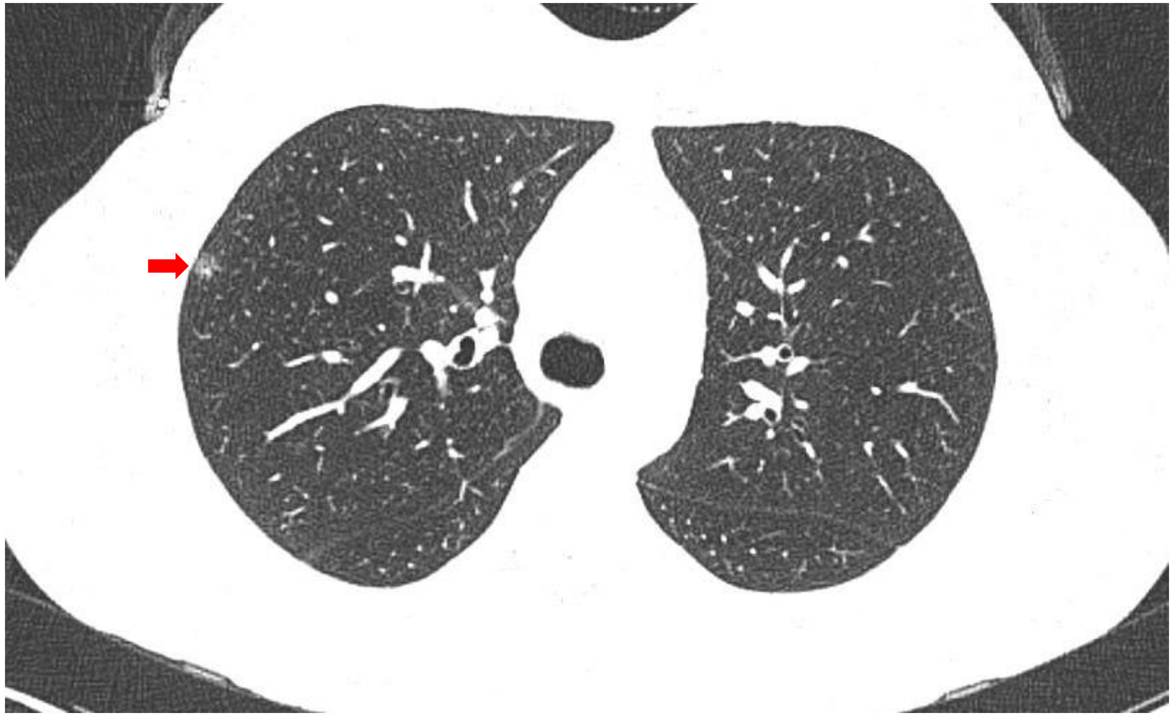

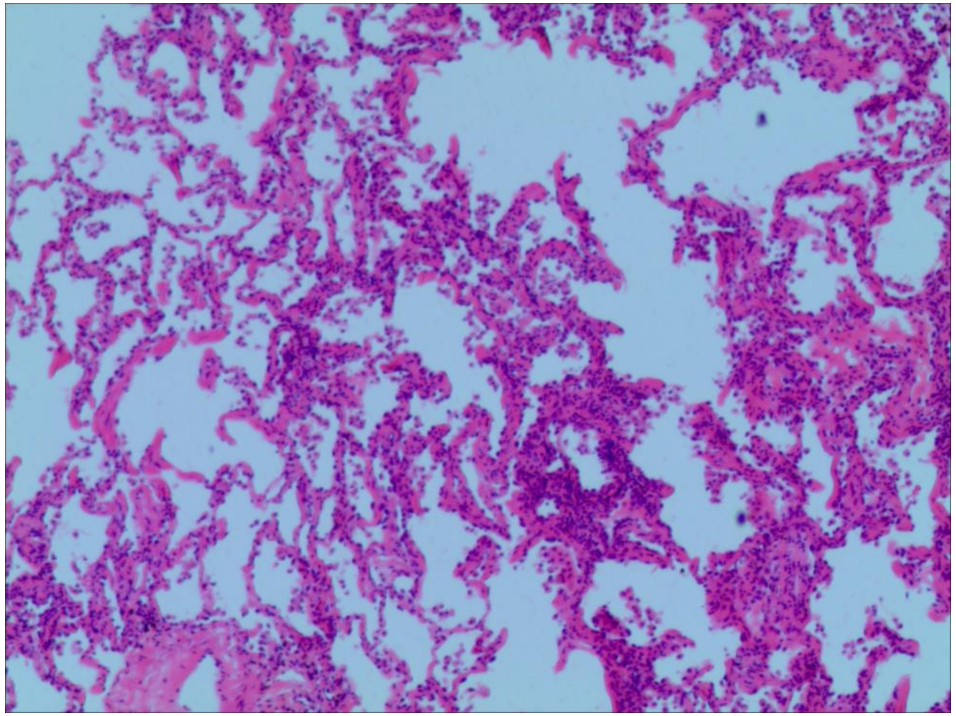

WL-10 CT (left) and IHC (right)

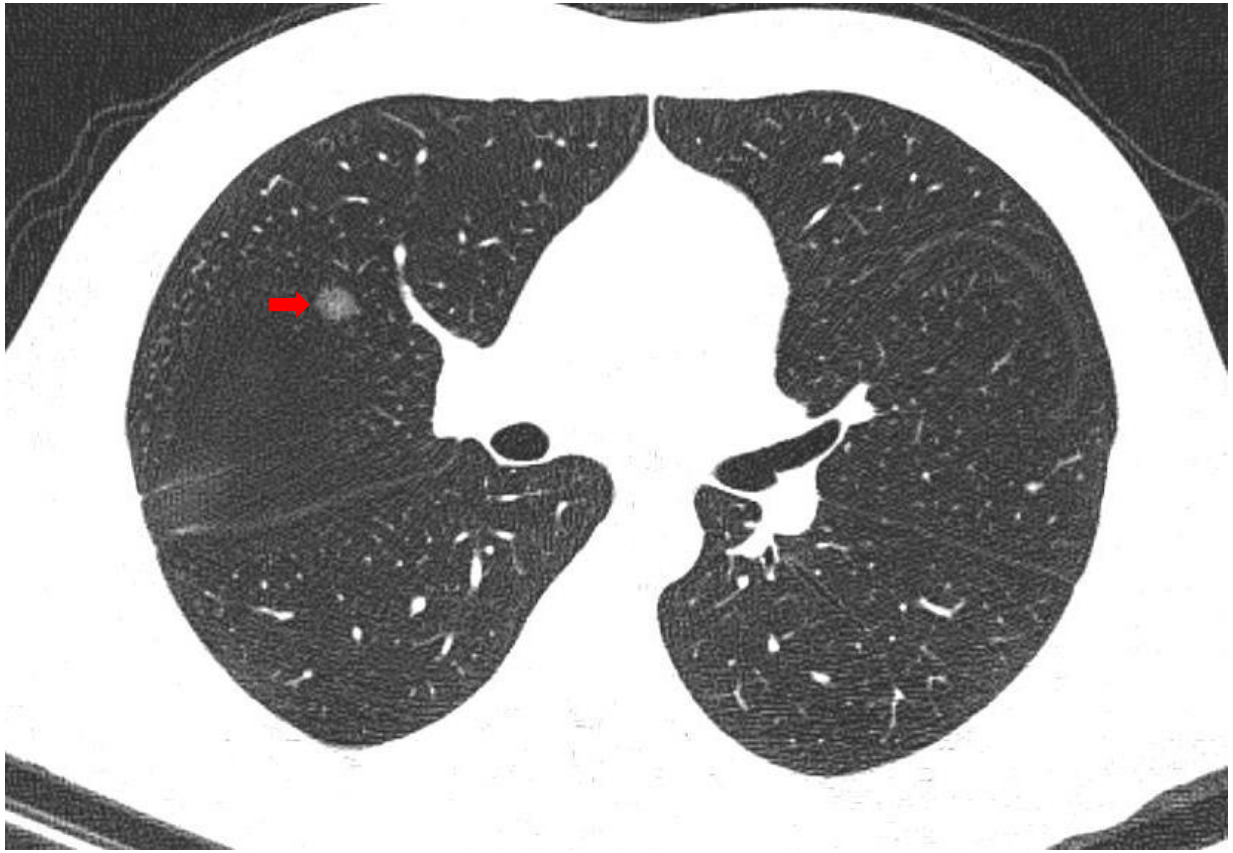

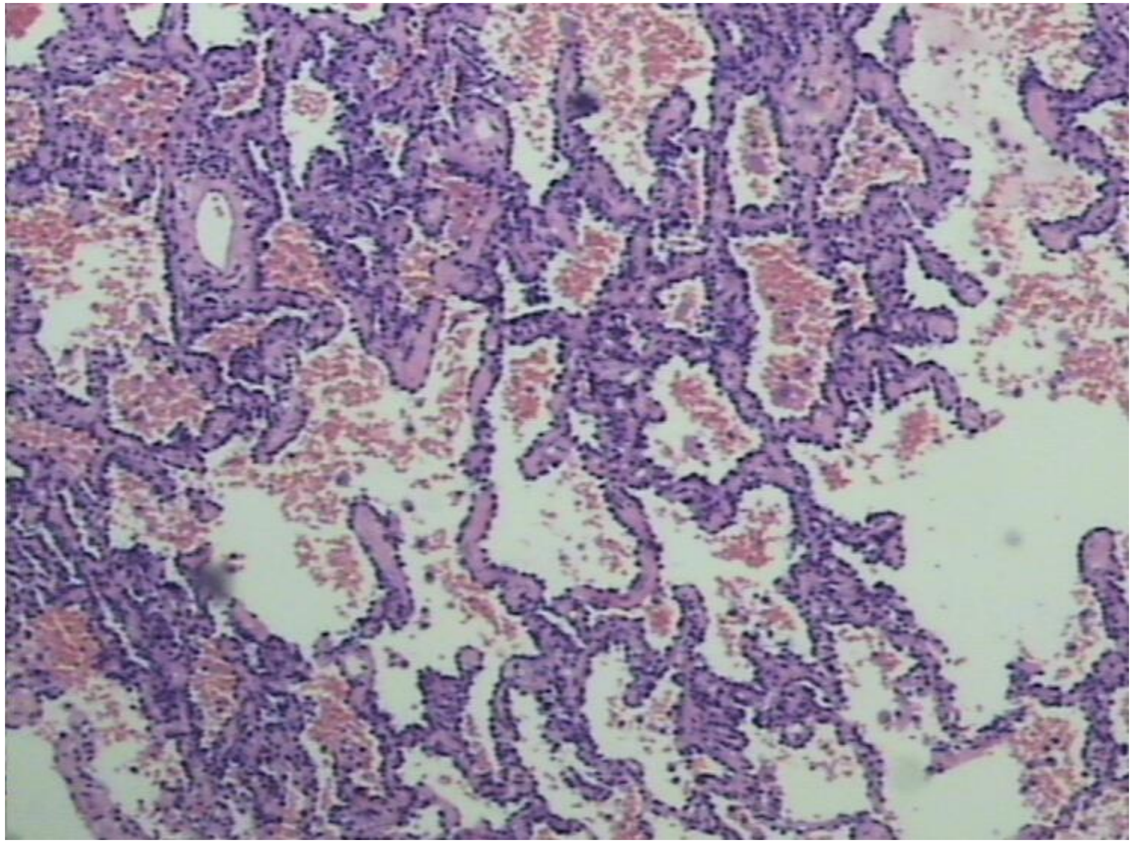

WL-11 CT (left) and IHC (right)

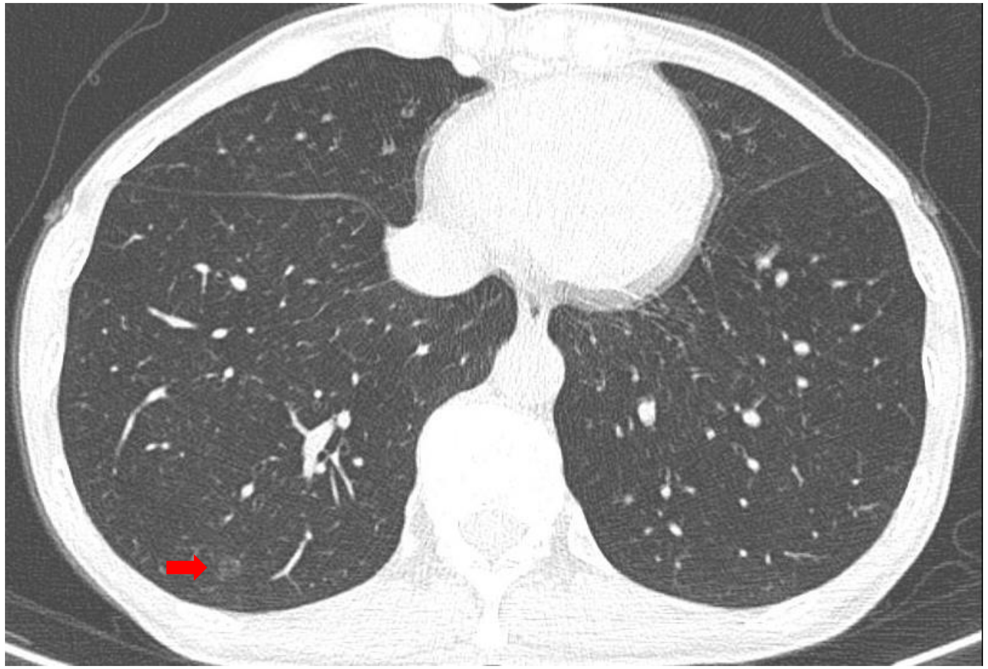

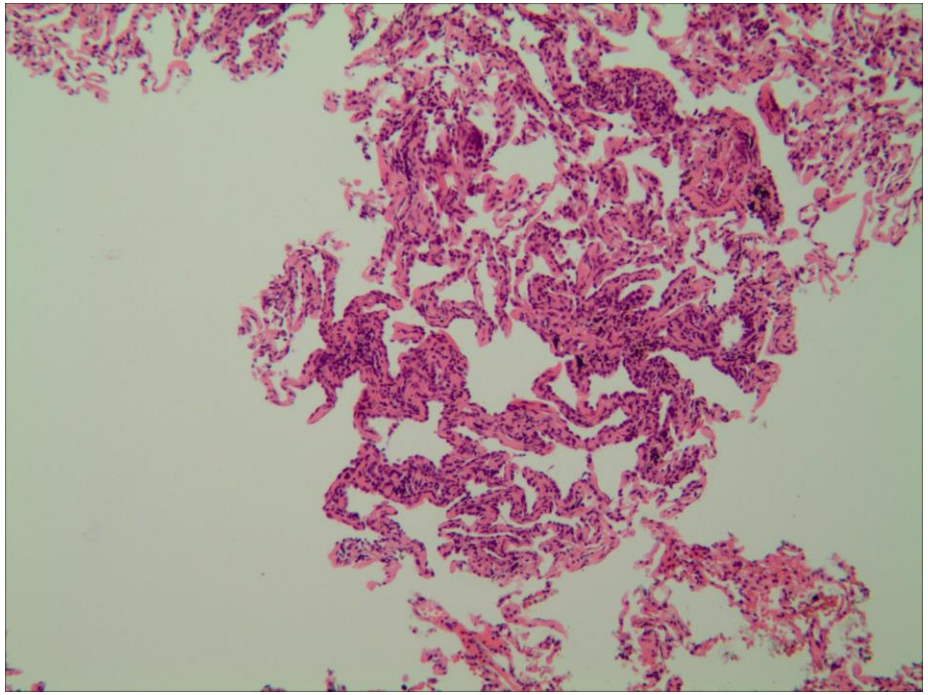

WL-12 CT (left) and IHC (right)

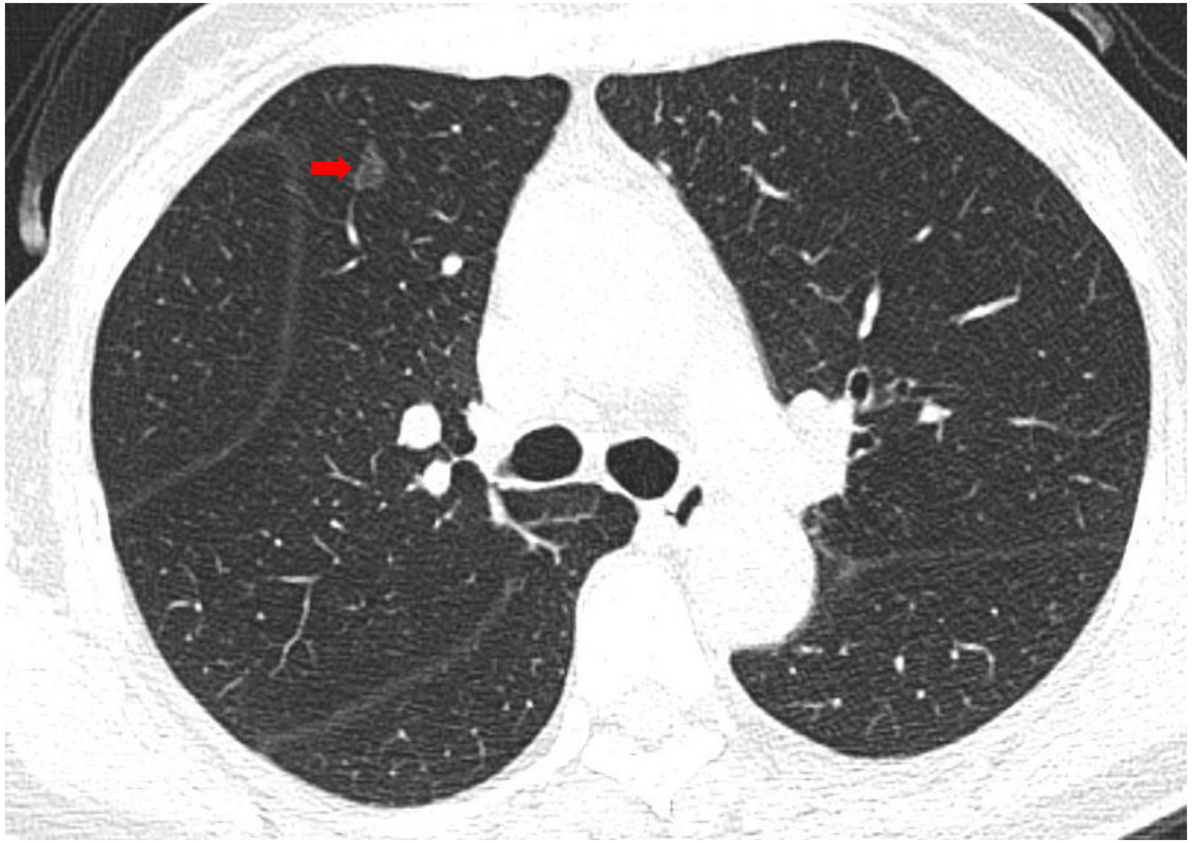

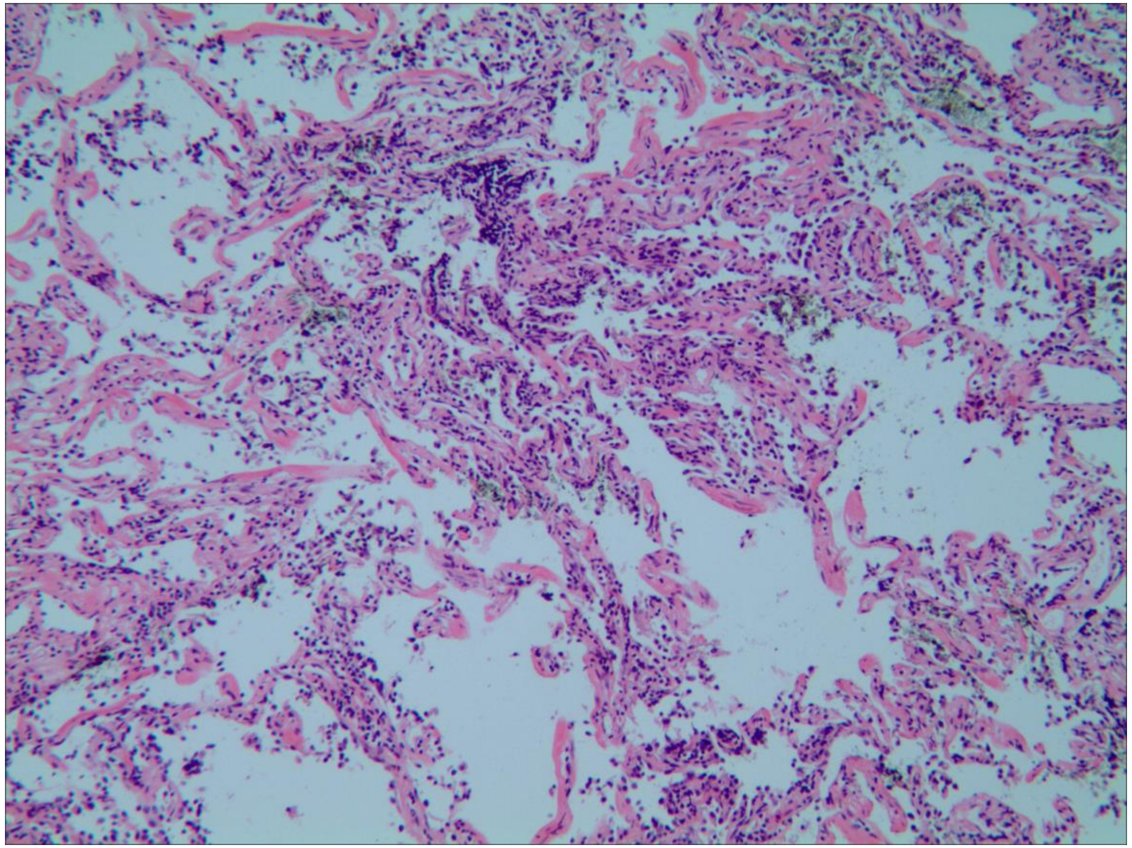

WL-13 CT (left) and IHC (right)

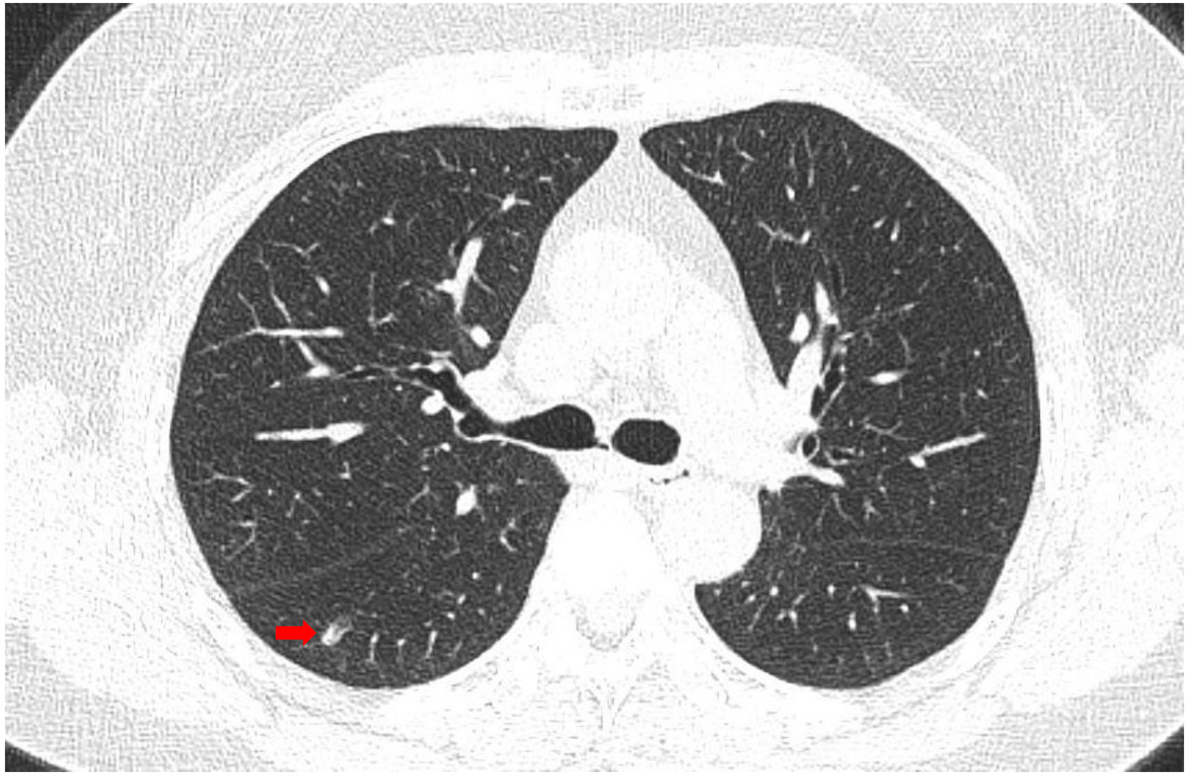

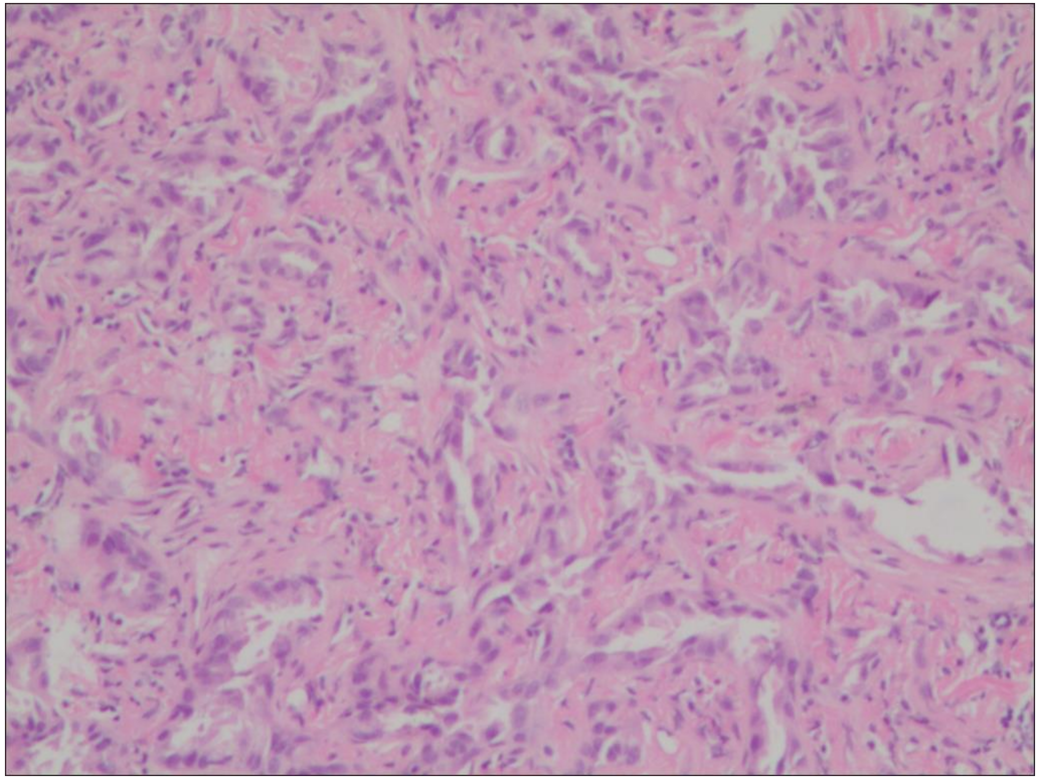

Supplement: Supplemental Information 2 — Computed tomography and immunohistochemistry images of patients [file peerj-09-12048-s002.pdf]
